# The influence of health literacy on how preschool educators and parents perceive the continuous development of health competencies within the framework of sustainability: Lithuanian case

**DOI:** 10.3389/fpubh.2024.1489816

**Published:** 2025-01-08

**Authors:** Laura Daniuseviciute-Brazaite, Lina Draudviliene

**Affiliations:** ^1^Faculty of Social Sciences, Arts and Humanities, Kaunas University of Technology, Kaunas, Lithuania; ^2^Ultrasound Research Institute, Kaunas University of Technology, Kaunas, Lithuania

**Keywords:** health literacy, preschool educators, parents, health competencies, framework of sustainability

## Abstract

Preschool education is one of the most important priorities of modern educational policies and the basis of lifelong learning. Health-literate educators and parents are better equipped to instill sustainable health practices in young children. Therefore, it is important to examine health literacy and determine how preschool educators and parents perceive the continuous development of health competencies within the framework of sustainability. The quantitative survey results revealed the dominant levels of general health literacy among respondents: problematic (31.1% vs. 33.5%) and sufficient (39.8% vs. 43.3%) for parents and teachers, respectively. Parents’ attitudes toward the development of children’s health competence within the framework of sustainability in preschool showed that parents and educators understand that their role is one of the most important factors in strengthening children’s health competence. However, most respondents spend only 2 h being active with children on healthy lifestyles (61.5% vs. 55%) for parents and teachers, respectively. A moderate correlation was found between health literacy and the weekly time spent developing children’s health competencies (*r* = 0.526). In addition, educators and parents who always or very often focus on the development of children’s health competence seek to plan daily activities in a way that would enhance health promotion (*r* = 0.463). Within the framework of sustainability, this influence becomes even more pronounced as sustainable health education aims to instill long-lasting, holistic health practices that benefit individuals and communities over time.

## 1 Introduction

Many countries in the World Health Organization (WHO) European Region have already addressed the learning of health knowledge, skills and competencies, attitudes, and behaviors in the context of health promotion and education efforts in schools. Health literacy is not synonymous with health promotion or education and will not replace them (1). Rather, it can be used in an action-oriented manner to address health misinformation, solve health problems, and promote health and well-being (1). The infodemic has shown how fast and at what magnitude misinformation and fake news about coronavirus and COVID-19 travel through the internet and social media platforms. Health literacy will help children and adolescents distinguish between trustworthy and false information and empower them to identify and avoid fake news (2). Higher levels of health literacy in children and adolescents have been associated with healthier behaviors and better health outcomes and status (3), making health literacy an important goal for health and education interventions early in life. Health literacy directly contributes to helping children, adolescents, and young people grow into critical thinkers and problem-solvers, as well as being ethically responsible, autonomous, and independent. It helps them become lifelong learners and empowered citizens, able to make informed decisions about their own lives and health and that of others (4). Many different literacy concepts from various research perspectives target these competencies. However, most of these (e.g., health literacy, environmental literacy, eco-ecological literacy, and sustainability literacy) remain within their inherent perspectives and disciplines (5). Some concepts (e.g., climate, health literacy, and environmental health literacy) reflect interdisciplinary approaches that link environmental and health perspectives (6). Education for planetary health could be one of the key levers in the much-needed civilizational turn toward a sustainable and healthy future.

A previous study (7) showed that educators lack information, knowledge, and competencies that would help develop children’s health competencies. According to Tandon’s (8) research, even 76% of preschool-age children spend about 35 h in preschool education institutions per week; therefore, educators are responsible for childcare, which plays an important role in developing health competence. Earlier research has shown that educators often lack knowledge on how to develop children’s health by preserving and strengthening competence and healthy living habits to form their health literacy (7). In addition, preschool children depend on their parents’ decisions to solve health problems, but they can also suffer when their parents have insufficient health literacy knowledge and skills (9, 10).

Health and education are linked inextricably. Delivering health literacy through schools has many benefits for society, such as economic and social growth, health behavior and outcome-related improvements, and academic and education-related benefits across life courses (11). A paradigm known as the Whole School Approach encourages entire school communities to advance fairness, health, and well-being in education (12). By promoting health through this link, the UNESCO Chair advances health not only as a physical education issue but also as a vital, integrated component of high-quality education, creating resilient, well-rounded children who are prepared for success in the classroom and in their personal lives.

Understanding is also important, that parents have the primary role and duty for health education since they decide what takes place at home. Nothing will change in terms of health literacy as long as parents do not collaborate or co-create these lessons in preschool education institutions. It is worth noting that the health education process in preschool educational institutions has not been distinguished by activity, which might be related to the different preschool education pedagogues’ understanding of health development. Thus, it is important to analyze how preschool educators and parents understand health literacy, how they value it, and how they value its significance in developing children’s health.

Therefore, the main research aim was to examine health literacy and how preschool educators and parents perceive the continuous development of health competencies within the framework of sustainability. More specifically, first to quantify the level of health literacy among educators and parents of preschool aged children, including their ability to identify, understand, and appraise health information. Second, we try to identify health-related skills perceived as challenging and document disparities in parental health literacy based on sociodemographic factors. Third and finally, we aimed to document the implications of parental health literacy on children’s health behaviors and outcomes.

## 2 Methods

**Figure 1:**
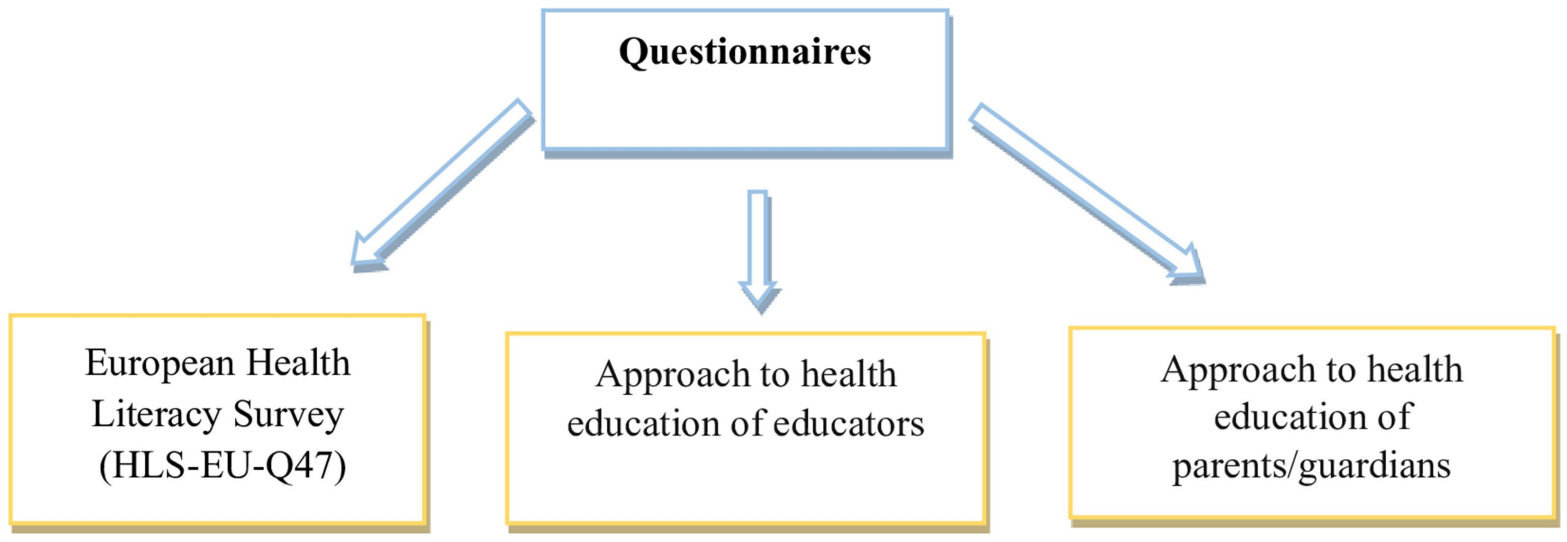
The questionnaires of preschool educators and parental health literacy in Lithuania through the approach of the implications for children’s health.

The design of the study includes three stages and different purposes: In the first stage, the purpose was to determine the health literacy levels of parents/guardians and educators using the European Health Literacy Survey Questionnaire (HLS-EU-Q47). In the second and third stages, the attitudes toward health education of parents/caregivers and educators were determined (Figure 1).

The HLS-EU-Q47 questionnaire was completed by 195 parents/guardians (153 females, 78.46%; 42 males, 21.54%). The mean age of the parents and guardians was 36 years (standard deviation [SD]-5.9, range 21–55) years old. The educational levels of the parents and guardians were relatively high (76.4%). The questionnaire was completed by 175 educators (162 females and 13 males). The teachers’ average age was 45.2 (standard deviation [SD] = 11.2). The respondents’ sociodemographic characteristics are presented in Table 1.

**Table 1:** Sociodemographic characteristics of the respondents.

No			Parents/guardians *N*	%	Average (x ± SN)	Educators *N*	%	Average (x ± SN)
1	Gender	Females	153	78.4		162	92.5	
		Males	42	21.5		13	7.4	
		In all	195	100		175	100	
2	Age	Females	153		35.9 ± 17.9			46.4 ± 23.5
		Males	42		37.2 ± 17.9			32.5 ± 22.3
		In all	195		36 ± 5.9			45.2 ± 1.2
3	Education	Secondary	13	6.6.		4	2.2	
		Professional	5	2.6		7	4	
		College	25	12.8		31	17.7	
		Bachelor	76	38.9		86	49.1	
		Master	73	37.4		44	25.1	

In the first stage, the long form of the HLS-EU-Q47 was developed based on a conceptual model of health literacy by Sorensen (5). The integrative model addresses the competencies of accessing, understanding, appraising, and applying health-related information for health promotion, disease prevention, and healthcare (5). A general-health literacy score that includes all items and three distinct sub-dimension scores covering health care, disease prevention, and health promotion are each used to represent a sub-dimension of the complete health literacy concept (13). It consists of three scales that help respondents to self-assess health literacy in the areas of healthcare (16 statements), disease prevention (15 statements), and health promotion (16 statements). Answers were given on a 4-point Likert scale (1 = very difficult, 2 = quite difficult, 3 = quite easy, 4 = very easy), leaving respondents with the option not to answer (the fifth answer option: I do not know). HLS-EU-Q47 scores were transformed into a unified metric scale with a minimum of 0 and a maximum of 50 points. A minimum of 0 points represents the least possible parental health literacy, and a maximum of 50 points represents the highest possible score (14). Based on the calculated health literacy index, four levels of health literacy were suggested: (1) 0 to 25 points, insufficient; (2) 25 to 33 points (problematic); (3) 33 to 42 points (sufficient); and (4) 42 to 50 points (very good) (1).

In the second and third stages, the purpose was to assess the values of preschool educators and parents toward the development of health competencies, which were compiled based on a literature review (15–18). The estimated values will help to identify health-related skills perceived as challenging and to document the implications of health behaviors and outcomes.

Questionnaires on values toward health education and sociodemographic characteristics were created for this study. The questionnaire consisted of 19 questions divided into five blocks.

- The first block of questions was intended to determine parents’ demographic data (sex, age, education, and sex of the child) and parents’ focus on health, which allowed to understand their initial opinions on this issue. Understanding is also important, that parents have the primary role and duty for health education since they decide what takes place at home. Nothing will change in terms of health literacy as long as parents do not collaborate or co-create these lessons in preschool education institutions.
- In the second block, respondents were asked questions related to the influence of family on the development of children’s health competence.
- The third block was intended to learn about methods of developing health competencies and the main aspects of strategies, organizing, and implementing these methods.
- In the fourth block, parents were asked what tools and forms of health competencies were used and how often children develop health competencies in a childcare institution by preschool educators.
- In the fifth block, respondents were asked to indicate which mental, social, and physical competencies of health promotion were important in preschool education

The questionnaire for preschool educators consists of 18 questions, most of which were similar to the questions and response options provided to parents; however, some questions are specifically intended for preschool educators. The educational questionnaire was divided into five blocks.

- In the first block, respondents were asked about demographic data, such as gender, age, education, teaching experience, qualifications, and institution where they teach.
- The second and third blocks of questions included questions in the parents’ questionnaire, their statements, and their response options.
- In the fourth block, respondents were asked to estimate how often health education was provided during preschool education.
- In the fifth block, respondents were asked to indicate which mental, social, and physical competencies of health promotion were important in preschool education.

The internal reliability of the questionnaires of preschool educators’ and parents’ attitudes toward the development of health competence was determined using the Cronbach-alpha coefficient (*α*) of the internal consistency of the test. It was found that the internal reliability of the questionnaire on parents’ and educators’ attitudes toward health education was sufficient because the value of Cronbach’s alpha coefficient was not lower than the minimum desired value of 0.7. Coefficient values for healthcare questions range from 0.79 to 0.80, enhancement coefficient values for disease prevention questions range from 0.58 to 0.67, and values for health promotion questions range from 0.68 to 0.79.

All procedures performed in this study were in accordance with the ethical standards of the institutional and/or national research committee and the 1964 Helsinki Declaration and its later amendments or comparable ethical standards. The study was approved by Regional Ethical Review MNL-KIN(B)-2019–212. Informed consent was obtained from all participants involved in the study.

Statistical analysis was performed using the SPSS software. Descriptive statistics were used to describe the distribution of the participants by sex, age group, education, and literacy level. The arithmetic mean and standard deviation (SD) were determined for comparison. Questionnaire research data were processed using percentage analysis, and the reliability of answers between research groups was calculated using the *χ*^2^ criterion. Frequency tables of characteristics were used to compare the attitudes of parents and educators toward the importance of physical, mental, and social health education. Interrelationships were assessed using Pearson’s correlation analysis. The confidence levels were set at 95% and thus differences with *p* < 0.05 were considered statistically significant.

## 3 Results

### 3.1 Health literacy from health care perspective

The responses of parents and educators to the health literacy questionnaire are presented in three tables according to the three determined blocks of questions divided by the topic above. The health literacy questionnaire started with the healthcare scale, and the results are presented in Table 2.

**Table 2:** Health care questions scale of parents and educators (%).

No	On a scale from very easy to very difficult, how easy would you say it is to …	Respondents	Very difficult	Difficult	Easy	Very easy	I do not know, I cannot answer
1	Deciding on the pros and cons of possible treatment options?	Parents	0.73	9	**57.42**	29.68	3.16
		Educators	1.47	15	**47.12**	27.57	2.47
2	Find information on treatments of illnesses that concern you?	Parents	1.70	16.55	**61.56**	17.52	2.68
		Educators	3.45	10.53	**69.34**	15.34	1.34
	
3	You decide that you may need another opinion from another doctor	Parents	1.22	14.84	**56.45**	21.65	5.84
		Educators	1.32	10.35	**66.34**	16.74	5.25
4	Find out where to get professional help when you get sick (e.g., doctor, pharmacist, psychologist)?	Parents	0.97	12.90	**47.45**	35.77	2.92
		Educators	1.22	7.70	**47.69**	41.69	1.7
5	Find out what to do in case of a medical emergency?	Parents	1.22	7.79	**47.69**	41.61	1.7
		Educators	1.95	8.76	**46.96**	37.96	4.37
6	Do you understand the information in the leaflets that come with the medicines?	Parents	1.95	8.76	**46.96**	37.96	4.38
		Educators	1.45	11.34	**53.12**	30.45	3.64
7	Find out where to get professional help when you are ill?	Parents	1.70	12.65	**50.36**	30.66	4.62
		Educators	1.45	13.56	**51.78**	28.45	4.69
8	Understand your doctor’s or pharmacist’s instruction on how to take prescribed medicine?	Parents	0.73	4.14	35.28	**58.88**	0.97
		Educators	0.56	3.34	33.23	**61.21**	1.56
9	Judge how information from your doctor applies to you?	Parents	0.49	8.03	**53.04**	33.58	4.87
		Educators	0.34	7.68	**57.34**	29.46	5.34
10	Judge the advantages and disadvantages of different treatment options?	Parents	1.46	15.09	**53.77**	22.38	3.30
		Educators	1.34	16.47	**56.36**	21.95	3.87
11	Judge when you may need to get a second opinion from another doctor?	Parents	0.97	15.57	**51.82**	25.55	6.08
		Educators	1.75	17.78	**58.74**	12.74	8.98
12	Judge if the information about illness in the media is reliable?	Parents	2.43	14.84	**52.80**	21.17	8.76
		Educators	3.47	15.45	**55.52**	17.58	7.98
13	Use information the doctor gives you to make decisions about your illness?	Parents	0.49	7.79	**58.64**	27.25	5.84
		Educators	1.20	6.78	**59.99**	27.48	4.56
14	Follow the instructions on medication?	Parents	0.73	5.84	43.31	**48.18**	1.95
		Educators	1.45	4.10	40.89	**52.78**	0.78
15	Call an ambulance in an emergency?	Parents	1.46	13.38	35.52	**41.85**	7.79
		Educators	1.87	12.25	32.38	**45.25**	8.25
16	Follow instructions from your doctor or pharmacist?	Parents	0.73	5.60	41.85	**51.09**	0.73
		Educators	0.52	6.10	42.01	**50.25**	1.12

The answers obtained indicate that most respondents understand healthcare questions quite easily. The distribution of answers to questions as very easy was, “to understand your doctor’s or pharmacist’s instruction on how to take prescribed medicine?” (58.8% parents, 61.2% educators), “follow instructions from your doctor or pharmacist?” (51.1% parents, 50.3% educators), “follow the instructions on medication?” (48.2% parents, 52.8% educators), and “call an ambulance in an emergency?” (41.9% parents, 45.3% educators). They reported that it was very easy to understand the information and perform the necessary actions. Only 16.6% of parents and 10.5% of educators found it difficult to find information about the treatment of diseases of interest and 14.8% vs. 10.4% found it quite difficult to know what to do in an emergency. Additionally, 14.8 and 15.5% reported difficulty in judging whether information about health risks in the media is reliable.

### 3.2 Health literacy from disease prevention

The second section of the questions was related to disease prevention, and the results are presented in Table 3.

**Table 3:** Scale of disease prevention questions of parents and educators.

No	On a scale from very easy to very difficult, how easy would you say it is to …	Respondents	Very difficult	Difficult	Easy	Very easy	I do not know, I cannot answer
1	Find information about how to manage unhealthy behavior such as smoking, low physical activity and drinking too much?	Parents	0.57	6.61	**51.44**	35.63	5.75
		Educators	0.47	7.82	**53.58**	32.58	5.55
2	Find information on how to manage mental health problems like stress or depression?	Parents	1.72	22.70	**44.25**	25.86	5.46
		Educators	1.45	20.49	**47.85**	24.58	5.63
3	Find information about vaccinations and health screenings that you should have?	Parents	2.87	21.26	**47.99**	25.86	2.01
		Educators	2.41	20.65	**49.87**	26.53	0.54
4	Find information on how to prevent or manage conditions like being overweight, high blood pressure or high cholesterol?	Parents	1.15	15.80	**52.01**	26.15	4.89
		Educators	2.01	15.34	**55.25**	24.15	3.25
5	Understand health warnings about behavior such as smoking, low physical activity and drinking too much?	Parents	0.00	4.89	35.63	**54.31**	5.17
		Educators	0.5	5.25	33.26	**58.56**	2.43
6	Understand why you need vaccinations?	Parents	2.30	11.49	38.79	**42.24**	5.17
		Educators	3.52	12.25	37.56	**44.52**	2.15
7	Understand why you need health screenings?	Parents	0.86	5.17	40.80	**49.71**	3.45
		Educators	1.05	4.85	39.56	**52.18**	2.36
8	Judge how reliable health warnings are, such as smoking, low physical activity and drinking too much?	Parents	1.15	8.05	37.07	**50.57**	3.16
		Educators	1.82	6.47	38	**51.26**	2.45
9	Judge when you need to go to a doctor for a check-up?	Parents	1.15	7.18	**44.83**	44.25	2.59
		Educators	1.25	6.25	**45.23**	44.15	3.12
10	Judge which vaccinations you may need?	Parents	1.72	16.95	**40.80**	34.20	6.32
		Educators	1.25	5.15	**46.33**	44.15	3.12
11	Judge which health screenings you should have?	Parents	0.86	15.52	**45.40**	34.20	4.02
		Educators	0.82	14.25	**44.15**	36.20	4.58
12	Judge if the information on health risks in the media is reliable?	Parents	2.01	14.08	**45.69**	30.17	8.05
		Educators	1.85	15.50	**47.25**	29.15	6.25
13	Decide if you should have a flu vaccination?	Parents	4.31	15.52	**35.92**	**35.92**	8.33
		Educators	4.02	19.28	**36.20**	**33.25**	7.25
14	Decide how you can protect yourself from illness based on advice from family and friends?	Parents	3.16	16.95	**45.98**	24.43	9.48
		Educators	2.85	15.82	**45.96**	25.12	10.25
15	Decide how you can protect yourself from illness based on information in the media?	Parents	2.30	19.25	**45.40**	23.56	9.48
		Educators	2.01	18.25	**47.25**	22.68	8.81

The answers to the disease prevention questions were arranged in a manner similar to that for healthcare. Most respondents found it relatively easy to find information on overcoming unhealthy habits and preventing or controlling obesity, high blood pressure, or excessive blood cholesterol. Deciding when to see a doctor for a health check-up (49.7% parents, 52.2% educators), what vaccinations may be needed (42.2% parents, 44.5% educators), how to protect oneself from diseases (54.3% parents, 58.6% educators). Most of them understand the questions about understandable warnings about smoking, low physical activity, and excessive drinking very easily—54.3% parents, 51.3% educators (see Table 3).

### 3.3 Health literacy from health promotion

The third section answers questions about health promotion and is presented in Table 4.

**Table 4:** Scale of health promotion questions of parents and educators.

No	On a scale from very easy to very difficult, how easy would you say it is to …	Respondents	Very difficult	Difficult	Easy	Very easy	I do not know, I cannot answer
1	Find information on healthy activities such as exercise, healthy food and nutrition?	Parents	0.32	3.48	**48.42**	45.89	1.90
		Educators	0.56	2.85	**49.32**	44.25	3.02
2	Find out about activities that are good for your mental well-being?	Parents	0.95	11.71	**43.67**	41.46	2.22
		Educators	1.25	10.28	**47.25**	39.33	1.89
3	Find information on how your neighborhood could be more health-friendly?	Parents	8.23	**33.86**	28.34	18.99	9.49
		Educators	8.12	**39.80**	26.58	17.25	8.25
4	Find out about political changes that may affect health?	Parents	6.33	**33.86**	31.33	18.99	9.49
		Educators	5.10	**35.28**	30.25	19.25	10.12
5	Find out about efforts to promote your health at work?	Parents	5.06	**41.77**	26.27	20.25	6.65
		Educators	6.32	**42.25**	22.93	21.25	7.25
6	Understand advice on health from family members or friends?	Parents	0.32	6.96	**54.75**	33.54	4.43
		Educators	0.45	7.25	**57.25**	32.15	2.90
7	Understand information on food packaging?	Parents	3.48	19.62	**46.84**	26.27	3.80
		Educators	3.15	20.15	**48.25**	25.60	2.85
8	Understand information in the media on how to get healthier?	Parents	0.95	10.13	**52.22**	33.54	3.16
		Educators	1.12	11.25	**54.02**	31.49	2.12
9	Understand information on how to keep your mind healthy?	Parents	1.58	12.03	**52.22**	29.11	5.06
		Educators	1.64	11	**53.12**	30.12	4.12
10	Judge where your life affects your health and wellbeing?	Parents	1.90	16.77	**48.42**	24.05	8.86
		Educators	2.10	17.85	**50.12**	22.01	8.01
11	Judge how your housing conditions help you to stay healthy?	Parents	1.58	7.59	**51.27**	35.44	4.11
		Educators	1.85	6.25	**54.12**	34.66	3.12
12	Judge which everyday behavior is related to your health?	Parents	0.63	6.33	**51.90**	39.56	1.58
		Educators	0.82	6.85	**53.25**	37.56	1.52
13	Make decisions to improve your health?	Parents	1.58	15.82	**47.15**	33.23	2.22
		Educators	1.42	12.03	**49.12**	32.58	4.85
14	Join a sports club or exercise class if you want to?	Parents	7.28	23.10	**38.29**	27.53	3.80
		Educators	6.85	24.12	**39.58**	26.60	2.85
15	Influence your living conditions that affect your health and wellbeing?	Parents	6.65	**39.56**	28.48	22.47	2.85
		Educators	7.45	**40.85**	28.45	21.40	1.85
16	Take part in activities that improve health and well-being in your community?	Parents	9.18	**33.23**	30.70	18.99	7.91
		Educators	9.54	**34.15**	30.85	18.34	7.12

Unlike in the healthcare and disease prevention question blocks, in health promotion, preschool educators and parents find it quite difficult to find information on how the ward could be more health-friendly (33.9% parents, 39.8% educators) and find it quite difficult to learn about political changes that may affect health laws (33.8% parents, 35.3% educators), and a large part of the respondents (41.7% parents, 42.3% educators) find it rather difficult to learn about efforts to improve health at work (Table 4).

### 3.4 Health literacy from social determinants of parental and educators’ health literacy

The sociodemographic factors of parents and teachers in comparison with the indicators of health literacy are presented in Table 5.

**Table 5:** Sociodemographic factors of parents and teachers in comparison with indicators of health literacy.

	*r*	95% CI	F-criterion value, *p*-level	*N*
Parents				
Gender	−0.085	−0.096, 0.177	3.872; *p* > 0.05	195
Age	0.009	−0.191, 0.013	3.87; *p* > 0.05	195
Gender of the child	−0.146*	−0.301, −0.026	**3.342; *p* < 0.05**	195
Residence	−0.082	−0.190, 0.010	1.463; *p* > 0.05	195
Education	0.422**	−0.016, 0.187	**2.09; *p* < 0.05**	195
Educators				
Gender		2.39, 0.267	1.292; *p* > 0.05	175
Age	−0.051	−0.191, 0.013	0.618; *p* > 0.05	175
Pedagogical work experience	−0.079	−0.301, −0.026	0.906; *p* > 0.05	175
Residence	−0.082	−0.190, 0.010	0.101; *p* > 0.05	175
Education	0.413**	−0.016, 0.187	**2.76; *p* < 0.05**	175

A statistically significant difference was found between parents’ lower level of health literacy and children’s gender indicators (*r* = −0.146; *p* < 0.05) and parents’ level of education (*r* = 0.422; *p* < 0.05). No statistically significant differences were found when comparing the remaining sociodemographic factors with the parental health literacy indicators (see Table 5).

### 3.5 Prevalence of categories of parental and educators’ health literacy in the sample

**Figure 2:**
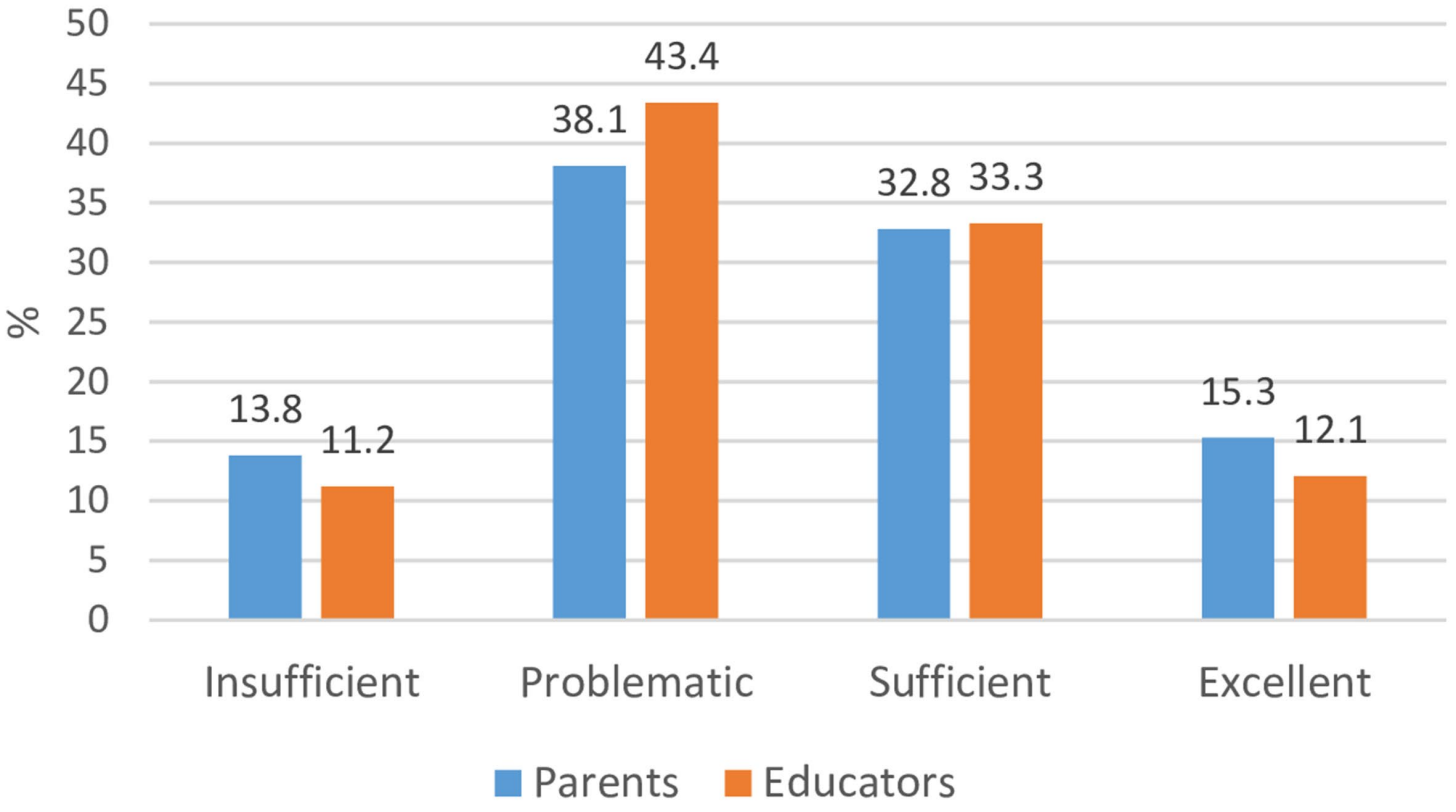
Health literacy levels of parents and educators (*χ*^2^ = 1.8; df = 3; *p* < 0.05). *Statistically significant difference between parents and educators.

Figure 2 showed that two levels of general health literacy were found to be dominant: problematic (31.1% of parents and 33.5% of educators) and sufficient (39.8% of parents and 43.3% of educators). Very good (15.3% of parents and 12.1% of educators) and insufficient (13.8% of parents and 11.2% of educators) belong to a small proportion of the respondents (see Figure 2). Statistically significant differences between health literacy levels were found (*χ*^2^ = 1.8; df = 3; *p* < 0.05).

### 3.6 Parental and educators’ health literacy and attitudes toward the development of health competence

The relationship between parents’ and educators’ health literacy and values toward health competence was analyzed (Table 6).

**Table 6:** Interrelationships between educators’ and parents’ health literacy and attitudes toward the development of health competence.

Respondents	Literacy level	Influence on living conditions	Planning health competence activities	Training in medical emergency	Contribution to child health promotion	Time devoted to health promotion
Factors influencing living conditions						
Parents	0.079	1	−0.068	−0.047	0.094	**0.144** ^*^
Educators	0.054	1	−0.062	−0.034	0.083	**0.121** ^*^
Planning daily health competence activities						
Parents	0.009	−0.068	1	0.141	**0.463** ^**^	**−0.225** ^**^
Educators	0.011	−0.064	1	0.135	**0.545** ^**^	**−0.285** ^**^
Training in dealing with medical emergency						
Parents	0.048	−0.047	0.141	1	0.124	**−0.256** ^**^
Educators	0.061	−0.052	0.186	1	0.111	**−0.289** ^**^
Contribution to child health promotion						
Parents	−0.004	0.094	**0.463** ^**^	0.124	1	**−0.413** ^**^
Educators	−0.009	0.082	**0.556** ^**^	0.152	1	**−0.456** ^**^
Time per week on health promotion competence						
Parents	−0.526**	**0.144** ^*^	**−0.225** ^**^	**−0.256** ^**^	**−0.413** ^**^	1
Educators	−0.621**	**0.187** ^*^	**−0.256** ^**^	**−0.278** ^**^	**−0.489** ^**^	1

Statistical confidence level of correlations: **p < 0.01; *p < 0.05.

A weak relationship was established between parents and educators, who were most influenced by factors influencing living conditions and the time devoted to developing health promotion (*r* = 0.144 parents, *r* = 0.121 educators; *p* < 0.05). It was established that those educators and parents who always or very often contribute to the development of children’s health competence aim to plan their daily activities in such a way that they are health-enhancing (*r* = 0.463 parents, *r* = 0.556 educators; *p* < 0.05). Parents and educators who planned daily activities for the development of children’s health competence spent more time dedicated to health competence development (*r* = −0.225 parents, *r* = −0.285 educators; *p* > 0.05). A weak correlation was found between the time spent on health competence and training on how to act in a medical emergency (*r* = −0.256; *p* < 0.05). A moderately strong correlation was found between health literacy and the amount of time spent per week developing children’s health competencies (*r* = 0.526 parents, *r* = 0.621 educators; *p* < 0.05). Parents (79.5%) answered that they are planning time to devote healthy lifestyle education, but spend little time being active with children on healthy lifestyles; 61.4, 21, and 17.4% of the respondents spent only up to 2 h, 3–5 h, and ≥ 5 h, respectively. Even 92% of the teachers’ answers to this question were positive.

## 4 Discussion

This study evaluated health literacy in early childhood by (1) investigating parents’ health literacy level and competence to improve the children’s health and health behavior, (2) investigating educators’ health literacy level and competence to improve the children’s health and health behavior, and (3) measuring competence and skills within the framework of sustainability.

Health literacy is a tool that can be utilized in an action-oriented manner to address health information, solve health problems, and improve health and well-being (19). Therefore, the determination of the health literacy levels among preschool educators and parents is one of the main aspects of the study. Thus, one key insight from the study is the variability in health literacy levels among preschool educators and parents. According to the health literacy evaluation, 33.5% of educators and 31.1% of the parents had problematic health literacy, 43.3% of educators and 39.8% of parents—sufficient health literacy. A social gradient in low health literacy is confirmed by research on adult, child and adolescent health literacy, which reveals that it is distributed unevenly among sociodemographic groups in Europe (19). For many years, the WHO has emphasized the relevance of health literacy in schools, including early promotion through health education and curriculum development (20), linking health literacy with and embedding it within whole-school approaches (21), highlighting that the mutual benefits for the education sector and society are linked to addressing health literacy in schools (11). This highlights the need for targeted interventions that address specific gaps in understanding and competencies.

According to the health literacy evaluation as problematic and sufficient, these characteristics were related to the area of disease prevention, where educators pointed out that it was fairly difficult to learn about efforts to improve health at work (41.8%), changing living conditions affecting health and well-being (drinking or eating habits, exercise, etc.; 39.6%), to participating in community activities that improve health and well-being (33.2%), to look for information on how their municipality could be more conducive to health (reduction of noise and pollution, creation of green spaces, development of leisure facilities, etc.; 33.9%), and to learn about policy changes that may affect health (laws, new health screening programs, etc.; 33.9%). The findings suggest that while educators play a pivotal role in shaping children’s health competencies, they often lack adequate resources, training, and knowledge. This supports earlier studies that found educators feel underprepared to incorporate health literacy into their teaching practices (7). Addressing these gaps could empower educators to actively engage in health literacy development, thereby enhancing their ability to instill critical health behaviors and attitudes in young children.

As mentioned earlier, our survey results showed that 31.1% of parents had problematic and 39.8% of parents—sufficient health literacy. The parental role in health literacy development is significant, as children’s health behaviors are heavily influenced by parental decisions and actions. The results reveal that parents’ health literacy levels can vary based on sociodemographic factors, suggesting that tailored approaches may be needed to address disparities. For instance, parents with lower health literacy may struggle to identify and act on credible health information, potentially compromising their children’s health outcomes. This finding aligns with prior research emphasizing that children often suffer when their parents lack adequate health literacy skills (9, 10).

Studies conducted in Canada (22), the United States (11), and Europe (16) show that health literacy is influenced by age, education, income, and perceived social status (16). In our study, the results did not show that age affected health literacy, but a dominant problematic level of health literacy was found in persons with secondary and vocational education. Also, respondents with master’s degrees had sufficient (42.3%), problematic (27.9%), insufficient (8.7%), and particularly proficient levels (21.2%) of health literacy.

The correlations between parents’ and educators’ health literacy and their subjective health confirm that individuals with higher health literacy tend to have better subjective health and quality of life. The correlation between education and health literacy is in line with previous empirical research (5) and with theoretical models of health literacy (2). In addition to personal skills and abilities, including knowledge, motivation, and competencies to deal with health information, the demands of the needed evidence, as well as the perceived and actual complexities of the health system, also influence when explaining self-perceived health literacy. Similar results were found by Spanish researchers who found that even though Health Education is included in the Early Childhood Education curriculum, teachers do not manage to develop it effectively (23). In addition, the child is often seen as a passive recipient of information rather than an active factor in promoting their family’s health (24). Researchers have highlighted this as an important gap in health education. Parents make decisions regarding their children’s health. Parents and caregivers participated in the interventions in a few of the research examined it was determined that children’s habits are closely linked to the health literacy and tooth brushing practices of their parents (25).

The results showed that parents and educators spend up to 2 h a week developing and strengthening children’s health competence; from 3 to 5 h, 21% of parents and 32.5% of educators spend time developing health competence. The education sector and schools offer an ideal setting for facilitating and enhancing the health literacy of children and adolescents as it reaches all school-aged children (26). School settings allow for the long-term implementation of health promotion activities and health education measures to strengthen health literacy sustainably. Activities can be designed to meet the needs and demands of different age groups and children with special educational needs and to reflect issues related to gender, diversity, ethnicities, and cultural and social (minority) groups (26). It can be reasonably argued that the role and importance of preschool teachers in health education remain central. The research confirms that the majority of the respondents stated that the role of the teacher is very important or important (95.4%). This also correlates with the results of other studies (27, 28).

The findings of this study underscore the critical role of health literacy as a foundation for fostering healthier behaviors and outcomes in children and adolescents, as well as its significance in empowering educators and parents to contribute to sustainable health education. The results align with the WHO’s emphasis on integrating health literacy into health promotion and education efforts within schools, demonstrating its potential to combat misinformation and equip individuals with the skills to navigate health challenges effectively. Thus, the working closely with schools, administrators, teachers, educationalists, educational personnel and parents to integrate health literacy into the curriculum, school culture, and classroom will help to develop into critical thinkers and problem solvers, morally upright, self-sufficient, lifelong learners, and capable citizens who can make wise decisions regarding their own and others’ health.

## 5 Conclusion

After assessing the health literacy of the parents and teachers, the health literacy level was determined to be problematic and sufficient. Higher education levels were found to be associated with higher levels of health literacy. After evaluating the values of preschool educators and parents toward the development of children’s health competence, it was found that both groups equally understood the importance of their roles in the development of children’s health competence. However, most parents devote only up to 2 h per week to the development of this competence, which causes difficulties in the child’s education process. A moderately strong correlation was found between health literacy and the time spent per week on developing children’s health competencies. Educators and parents who always or very often contribute to the development of children’s health competence reported planning their daily activities to enhance health. While the sample is not representative of the entire country, the collected data provide a robust insight into the relationship between parental health literacy and children’s health in Lithuania.

Therefore, additional research is needed to further explore the relative contribution of parental and educators’ health literacy. It should also be noted that the respondents may exaggerate or underestimate their health literacy skills and related activities, the use of self-reported data raises the possibility of bias. Additionally, the study’s depth is limited by its exclusive reliance on quantitative approaches, as qualitative insights could offer a deeper understanding of the difficulties parents and educators confront in encouraging health literacy.

## Data Availability

The original contributions presented in the study are included in the article/supplementary material, further inquiries can be directed to the corresponding author.
